# Plasma cell-free RNA profiling of Vietnamese Alzheimer's patients reveals a linkage with chronic inflammation and apoptosis: a pilot study

**DOI:** 10.3389/fnmol.2023.1308610

**Published:** 2023-12-21

**Authors:** Thien Hoang Minh Cao, Anh Phuc Hoang Le, Tai Tien Tran, Vy Kim Huynh, Bao Hoai Pham, Thao Mai Le, Quang Lam Nguyen, Thang Cong Tran, Trang Mai Tong, The Ha Ngoc Than, Tran Tran To Nguyen, Huong Thi Thanh Ha

**Affiliations:** ^1^School of Biomedical Engineering, International University, Ho Chi Minh City, Vietnam; ^2^Vietnam National University, Ho Chi Minh City, Vietnam; ^3^Department of Physiology, Pathophysiology and Immunology, Pham Ngoc Thach University of Medicine, Ho Chi Minh City, Vietnam; ^4^Department of Neurology, Faculty of Medicine, University of Medicine and Pharmacy at Ho Chi Minh City, Ho Chi Minh City, Vietnam; ^5^Department of Neurology, University Medical Center, Ho Chi Minh City, Vietnam; ^6^Department of Geriatrics, Faculty of Medicine, University of Medicine and Pharmacy at Ho Chi Minh City, Ho Chi Minh City, Vietnam; ^7^Department of Geriatrics and Palliative Care, University Medical Center, Ho Chi Minh City, Vietnam

**Keywords:** Alzheimer's disease, cell-free biomarkers, RNA-sequencing, differential expression, co-expression, immune responses

## Abstract

**Introduction:**

Circulating cell-free RNA (cfRNA) is a potential hallmark for early diagnosis of Alzheimer's Disease (AD) as it construes the genetic expression level, giving insights into the pathological progress from the outset. Profiles of cfRNA in Caucasian AD patients have been investigated thoroughly, yet there was no report exploring cfRNAs in the ASEAN groups. This study examined the gap, expecting to support the development of point-of-care AD diagnosis.

**Methods:**

cfRNA profiles were characterized from 20 Vietnamese plasma samples (10 probable AD and 10 age-matched controls). RNA reads were subjected to differential expression (DE) analysis. Weighted gene correlation network analysis (WGCNA) was performed to identify gene modules that were significantly co-expressed. These modules' expression profiles were then correlated with AD status to identify relevant modules. Genes with the highest intramodular connectivity (module membership) were selected as hub genes. Transcript counts of differentially expressed genes were correlated with key AD measures—MMSE and MTA scores—to identify potential biomarkers.

**Results:**

136 genes were identified as significant AD hallmarks (*p* < 0.05), with 52 downregulated and 84 upregulated in the AD cohort. 45.6% of these genes are highly expressed in the hippocampus, cerebellum, and cerebral cortex. Notably, all markers related to chronic inflammation were upregulated, and there was a significant shift in all apoptotic markers. Three co-expressed modules were found to be significantly correlated with Alzheimer's status (*p* < 0.05; *R*^2^> 0.5). Functional enrichment analysis on these modules reveals an association with focal adhesion, nucleocytoplasmic transport, and metal ion response leading to apoptosis, suggesting the potential participation of these pathways in AD pathology. 47 significant hub genes were found to be differentially expressed genes with the highest connectivity. Six significant hub genes (*CREB1, YTHDC1, IL1RL1, PHACTR2, ANKRD36B, RNF213*) were found to be significantly correlated with MTA and MMSE scores. Other significant transcripts (*XRN1, UBB, CHP1, THBS1, S100A9*) were found to be involved in inflammation and neuronal death. Overall, we have identified candidate transcripts in plasma cf-RNA that are differentially expressed and are implicated in inflammation and apoptosis, which can jumpstart further investigations into applying cf-RNA as an AD biomarker in Vietnam and ASEAN countries.

## 1 Introduction

Alzheimer's disease (AD) is the most common type of dementia—accounting for 60–80% of dementia cases (Prince, 2015). Alzheimer's and other dementia-related disorders are recognized by cognitive impairment, and with its steady growth in the number of cases, it has become one of the greatest health concerns in the 21st century (Rasmussen and Langerman, 2019; Porsteinsson et al., 2021). In brief, AD is a progressive, irreversible disease accompanied by genetic anomalies and manifested in the stage of aging, which targets speech, cognitive processing, and predominantly, memory, jeopardizing the wellbeing and the quality of life of the patients (Hampel et al., 2017; Knopman et al., 2021). A 305-billion-dollar annual expense was recorded in the USA for AD and dementia-related disorders, and such cost was predicted to triple by 2050 (Porsteinsson et al., 2021). The presented statistics accentuate the current substantial economic stress on many parties (e.g., patients, families, or government) and highlight the massive medical burden in every nation. Until today, there have been no effective treatments for AD when it has exceeded the Mild Cognitive Impairment (MCI) stage. All ongoing efforts concentrate on improving early diagnostic methods for AD to impede the progression of the disease from an early stage (Rasmussen and Langerman, 2019).

However, diagnosing Alzheimer's disease (AD) remains a challenge thus far. Clinical symptoms include impairments in episodic memory, linguistic, executive, and visuospatial functions that overlap those of other dementia, which require advocacy from neuropsychiatric, imaging, and biological tests (Beach et al., 2012; Porsteinsson et al., 2021). Extensive assessments like magnetic resonance imaging (MRI) and positron emission tomography scans (PET) are labeled as expensive means, yet necessary for a probable diagnosis of AD (Houmani et al., 2018; Kim K. et al., 2020). These assessments, however, can be either incapable of detecting the presymptomatic stage of AD, which can occur decades ahead of the brain atrophy and disease onset, or overpriced. On the other hand, underlying AD pathology is associated with the accumulation of Amyloid-beta (Aβ) plaques and tau tangles. They are built up gradually through the AD continuum starting from the presymptomatic stage (Reitz et al., 2020; Surguchov et al., 2023). The premise has deviated the focus of academia to biomarkers residing in cerebrospinal fluid (CSF) and biofluids since there were strong correlations found between the markers and the etiology of AD (Suárez-Calvet et al., 2018; Twohig et al., 2018; Pais et al., 2020; Reitz et al., 2020). Presently, CSF Aβ42 and the Aβ42/40 ratio, CSF total tau, and phosphorylated tau are widely recognized as extensive tests for clinical AD diagnosis (Kerwin et al., 2022). However, this approach is costly and carries great health risks from the lumbar puncture procedure, which can not be applied as a screening routine for cognitive health and a means for early diagnosis. The current situation calls for an accurate, robust, and less invasive novel approach.

By providing an objective and quantitative measure of the progressing pathophysiology, biomarkers are considered a reliable criterion for AD. Through rapid advances in ultra-sensitive assays, AD-related markers can be detected in blood samples and contribute to developing less-invasive AD diagnoses. Previous studies have shown that blood-based immunoassays yield notable AUC: plasma Aβ42/Aβ40 (AUC = 0.8) (Palmqvist et al., 2019); Aβ42/Aβ40 combined with *APOE* genotyping or Nfl (~0.85–0.87) (Schindler et al., 2019); Plasma *p*-tau231 (AUC = 0.93) (Ashton et al., 2021). Compared to proteomic biomarkers, nucleic acid biomarkers possess several advantages. *Apolipoprotein E (APOE)* gene is one of the precedent nucleic acid hallmarks for AD, which expresses the APOE protein that maintains lipid homeostasis via lipid transport throughout the body (Liu et al., 2013; Raulin et al., 2022). In the central nervous system (CNS), cholesterol is delivered to neurons via communication between the APOE predominantly secreted by astrocytes and the APOE receptors—LDL receptors (Herz, 2009; Lane-Donovan and Herz, 2017). Notably, it has been well-established that the *APOE* gene is an important genetic risk factor for the pathology of AD (Raulin et al., 2022), and its polymorphism is the grave threat determinant of late-onset AD (Yamazaki et al., 2019). There are three polymorphic alleles of the *APOE* gene, including ε2, ε3, ε4, and their corresponding worldwide distributions are 8.4, 77.9, and 13.7% (Liu et al., 2013). From that, the ε4 carriers face a higher threat of AD than the homogeneous ε3 carriers. Particularly, AD risk increases 3–4 fold if carriers possess one ε4 allele and 9–15 folded-increased threat in two-ε4-allele carriers (Yamazaki et al., 2019). In addition to *APOE*, cell-free RNAs (cfRNA or extracellular RNAs) are promising biomarkers that can unravel the underlying etiology, pathology, and AD progression. CfRNAs are defined as RNA existing outside cells, bounded by exosomes, micro-vesicles, oncosomes, or similar lipid/protein complexes. Originating from different types of cells via either secretion or apoptosis, thanks to the extracellular vesicle encapsulation, cfRNA can be circulated in biofluids (plasma, urine, saliva, and cerebrospinal fluid) without being degraded by ribonucleases (Sadik et al., 2018; Gruner and McManus, 2021; Dellar et al., 2022; Le and Huong, 2022). CfRNAs can differentiate the control group from the disease-carrier group, as well as inform clinicians of the disease progress from the early stage, aiding disease screening and monitoring (Schwarzenbach et al., 2011; Bhatnagar et al., 2014; Burgos et al., 2014; Yan et al., 2020). Recent studies suggested multiple candidates, including the transcripts of *EEF2* and *RPL7* (AUC = 0.878), *PROK2, SLU7, LRRK2* (AUC = 0.83), *ABCA7* and *AKAP9* (AUC = 0.77) that are associated with the downregulation of multiple neurogenesis pathways such as GABA signaling and neurotransmission (Shigemizu et al., 2020; Toden et al., 2020). Analysis of RNA-biomarkers is also more feasible compared to proteomic biomarkers as they need to include post-translation in the picture. Due to these notable benefits, they are receiving attention as prominent biomarkers for AD.

In Vietnam and other neighboring low-middle-income countries (LMICs), diagnosing Alzheimer's disease, in general, remains an unsolved dilemma. In addition to the discussed shortcomings of current diagnostic means such as MRI, PET, and CSF-test, diagnosing AD in LMICs has to overcome two obstacles: limited medical resources and illiteracy (Hoi et al., 2010; Nguyen and Wilson, 2017). With the limited diagnostic capacity of primary care facilities, especially in rural areas, the role of medical questionnaires became vital for AD screening. However, results from questionnaires like MMSE and MoCA can be influenced by the patient's literacy (Nguyen et al., 2023). With the illiteracy rate of the Vietnamese population in rural areas reaching 18%, neuropsychiatric tests are no longer the appropriate resolution (Hoi et al., 2010). Therefore, it is essential to develop an alternative diagnostic approach that is both affordable and feasible for patients located in rural areas. One of the prominent resolutions is a blood test that targets circulating cfRNA, which has been extensively studied in previous works discussed above. By integrating novel blood-based cfRNA biomarkers, the cost of routine clinical assessments for AD can be reduced, and the test can be widely conducted. This is fundamental for LMICs, especially in rural areas where the medical facilities are scant and the illiteracy rate among elders is highly noted. In addition, these blood tests can precede conventional PET and MRI scans by reducing false negative results in the early stage, as well as providing insights into the disease's heterogenic pathology during the progression, which can consequently save patient's time and money (Cummings et al., 2019; Wang et al., 2023).

This study focuses on conducting a preliminary investigation of the differences in cfRNA transcriptomic profiles between Alzheimer's and cognitive normal cohorts in Vietnam. Through a combination of differential expression and co-expression analysis, we sought to identify which genes are key drivers of expression changes between the two groups, which are potentially relevant to the development and progression of Alzheimer's disease. We then examined whether the cfRNA transcripts significantly correlate with conventional measures of Alzheimer's disease severity (MRI and MMSE-score) to identify candidate cfRNA markers for the future development of a clinical blood-based test.

## 2 Materials and methods

### 2.1 Study design, participants, and IRB approval

**Figure 1:**
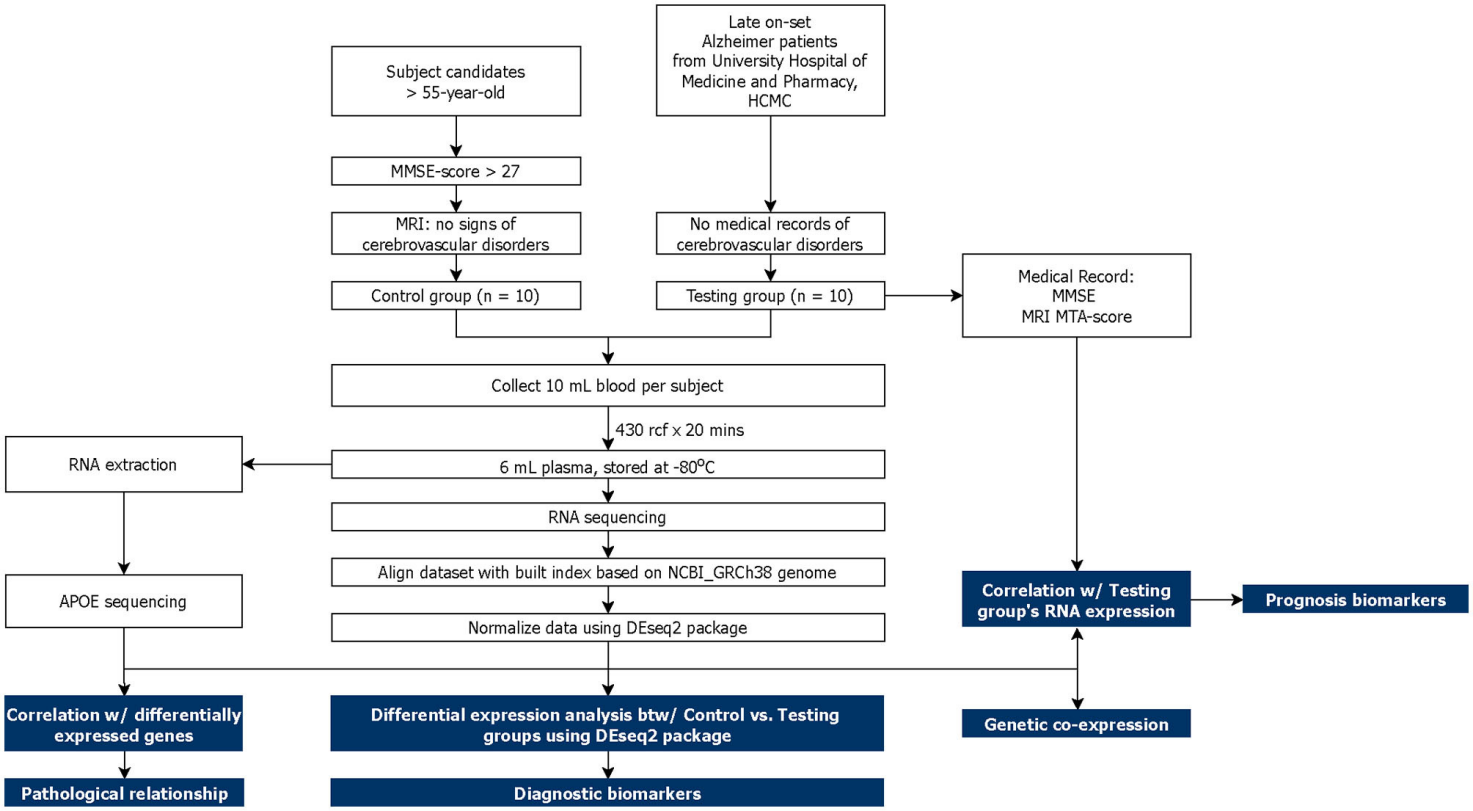
Schematic of study design. The main analysis and results are highlighted in blue.

This study was approved by the Institutional Review Board (IRB) of University Medical Center, Ho Chi Minh City (UMC-HCMC). A total of 20 subjects aged above 55 years old were recruited into two cohorts: Alzheimer-diagnosed cohort—AD (*n* = 10) and cognitive normal control cohort—CNC (*n* = 10). All subjects were thoroughly explained the terms and conditions of the experiment before signing a consent form. The AD subjects were chosen from the diagnosed AD patients at the UMC-HCMC without accompanying cerebrovascular and other neurodegenerative diseases. The CNC subjects were selected based on (1) MMSE score ≥ 27 (Folstein et al., 1975; He et al., 2022); (2) absence of memory complaints or any other cognitive symptoms; (3) no sign of neurological or psychiatric dysfunctions; (4) Clinical Dementia Rating (CDR) = 0 (Morris, 1993; Galvin, 2015). MRI images were captured for CNC subjects and used to select subjects without cerebrovascular diseases and neurodegeneration. Blood samples were then collected from the subjects and proceeded with plasma cfRNA sequencing and follow-up analyses that are described in Figure 1. Differential expression (DE) analysis was conducted between two cohorts to identify potential cfRNA diagnostic markers while prognostic markers were retrieved from the correlation between the testing cohort's transcriptomic counts and other medical records (MMSE and MRI MTA-score). Genetic co-expression and *APOE* traits were also included in this report.

### 2.2 Sample collection and RNA sequencing

Ten mL of blood was drawn from each subject into the Norgen cf-DNA/cf-RNA Preservative tube (#63950, Norgen Biotek, Canada) and centrifuged at 430 relative centrifugal force (rcf) for 20 min. The supernatant was collected, giving 6 mL plasma per subject. Both plasma samples and residual blood cells were then stored separately at −80°C for cfRNA sequencing and *APOE* genotyping. RNA sequencing was conducted by BGI Hongkong Tech Solution NGS Lab (BGI Genomics, Hong Kong) using the Nugene low-input RNA sequencing protocol and the DNA Ball Sequencing (DNBSEQ) platform. Poly-A enrichment depletes undesired ribosomal RNA (rRNA), leaving purified circulating messenger RNA (mRNA). After sequencing, the data were filtered by removing the adaptor sequences, contamination (polyX; N content ≥ 1%; read length < 100 bp), and low-quality raw reads.

### 2.3 APOE genotyping

According to manufacturer instructions, DNA extraction was performed on the remaining blood pellet using the Blood DNA Isolation Mini Kit (#46300, Norgen Biotek, Canada). The concentration and purity of the extracted DNA were assessed using a Nanodrop machine. *APOE* genotypes (*ApoE3* and *ApoE4*) were identified through allele-specific PCR. The primer sequences used are described in Table 1. The *APOE* isoforms E3 and E4 are defined by a single nucleotide polymorphism (T-to-C) at position 3937 (rs429358) of the *APOE* gene, which codes for the 112th amino acid of the resulting *APOE* protein. This mutation can be detected by PCR with *APOE* allele-specific primers, as described in previous studies (Seripa et al., 2006).

**Table 1:** Primer sequences.

**Primer**	**Direction**	**Sequence**
**Allele-specific primers for APOE-E3/E4**		
E3	F	CGGACATGGAGGACGTGT
E4	F	CGGACATGGAGGACGTGC
E3m	F	CGGACATGGAGGACGTTT
E4m	F	CGGACATGGAGGACGTTC
Common reverse	R	GCTTCGGCGTTCAGTGATTG
**Positive control primers**		
ACTB-F	F	GACGTGGACATCCGCAAAGAC
ACTB-R	R	CAGGTCAGCTCAGGCAGGAA

ACTB, actin beta housekeeping gene.

We initially employed two primers (E3 and E4) that were used for APOE genotyping in previous studies (Calero et al., 2009). To improve the discriminating power between the E3 and E4 allele, we designed two additional primers, E3m and E4m, which contain an additional single nucleotide mismatch before the mutation site (3′ end) (Bui and Liu, 2009). This increases the destabilizing power between the primers and a non-target template (e.g., the E3 primer with an E4 template), reducing the chance of a false positive amplification. One microliter of each DNA sample was added to a mixture of different primer pairs and a PCR Master Mix (Phu Sa Genomics, Vietnam). For each PCR reaction, an allele-specific primer was paired with the common reverse primer. Each reaction also includes a positive control primer pair (*ACTB-F* and *ACTB-R*), which amplifies a region in the *ACTB* gene. Because of the high GC% content of the target region, 5% DMSO was added to the PCR mixture to enhance amplification. The resulting amplicons were visualized through standard agarose gel electrophoresis (Supplementary Data 1).

### 2.4 RNA-sequencing data analysis

#### 2.4.1 Data preprocessing

All data preprocessing was conducted using R.v.4.2.2. Firstly, the raw sequencing data went through the “Built and align” stage. Genome Reference Consortium Human Build 38 (GRCh38) was downloaded from the NCBI datahub and input as the reference genome for the library index. The Rsubread (v.2.12.3) package was then installed to build the library index based on the reference genome, and raw sequencing data were aligned accordingly to the index. The feature counts function extracted raw counts data from the aligned sequencing data and exported the results into a text file. With each subject, the counts from two sequencing reads were summed up to extract a file of the total raw counts. The extracted file was then input for the sequential stage—“Preprocessing.” Function cpm from the EdgeR (v.3.40.2) package was used to normalize the raw counts' data into the counts per million reads mapped (cpm) unit. Five hundred and eighty-one genes with a threshold cpm > 0.5 in more than five sequenced samples of either cohort were kept for further analysis. The R scripts used to perform the following analyses can be found in our repository at https://github.com/miti08/VAN-R-scripts/.

#### 2.4.2 Differential expression analysis

Differential expression (DE) analyses were conducted on 581 kept genes to identify genes with significant differences in expression levels between the two investigated cohorts. The following packages were installed, respectively, into R to conduct DE analysis: BiocManager (v.1.30.20), DESeq2 (v.1.38.3), ggplot2 (v.3.4.1), limma (v.3.54.1), gplots (v.3.1.3), AnnotationDbi (v.1.60.2), org.Mm.eg.db (v.3.16.0), Glimma (v.2.8.0), RColorBrewer (v.1.1-3), ggrepel (v.0.9.3), EnhancedVolcano (v.1.16.0). The raw counts' data of kept genes were input to form a data matrix using DESeqDataSetFromMatrix. Differential expression (DE) analysis was conducted on the data frame using the DESeq function (Love et al., 2014). Log2foldchange (log2FC) and the adjusted *p*-value (adj.*p*) of analyzed genes were used to build the volcano plot using the EnhancedVolcano function. This step was conducted to visualize the distribution of selected genes with respect to the level of significance (adj.*p*) and level of difference (log2FC). The adj.*p* and log2FC of 88 significantly differential expressed genes (adj.*p* < 0.05) were exported into a dataset. Due to variance posed by long-term storage degradation, raw counts data of 136 significantly differential expressed genes were also normalized into the median of ratios using the DESeq2's counts function for better comparison. The adj.*p* and log2FC, and the normalized raw counts of 136 genes were used as input for heatmap visualization in Python v.3.8, using the seaborn.heatmap package (0.12.2). Confirmed to be circulated at a stable level in biofluids regardless of cognitive impairments, GAPDH was considered as the reference gene to confirm the reliability of the DE analysis in this study (Kim K. M. et al., 2020; Guennewig et al., 2021; Zhang et al., 2021). We hypothesized that the measured differences are reliable if the counts of GAPDH in the two cohorts were insignificantly different.

#### 2.4.3 Weighted gene co-expression analysis

Co-expression analyses were performed using the Weighted Gene Co-expression Network Analysis (WGCNA) package for R (Langfelder and Horvath, 2008). The filtered expression matrix of 581 genes was used as the input. The WGCNA package constructed a signed adjacency matrix based on Pearson correlations. From this matrix, hierarchical clustering and dynamic tree cutting were performed to identify gene clusters (modules) with strong co-expression. WGCNA then assigns arbitrary colors to each module (e.g., blue, yellow, green, etc.) for reference purposes. To identify modules of interest for further analysis, the module eigengenes (a measure of overall module gene expression) were correlated with clinical variables, including Alzheimer's disease status, age, and sex. Functional enrichment analysis was then performed on the modules of interest to elucidate the overall functional characteristics of each gene module. Intramodular analysis of the module genes was performed to calculate two key measures for each gene: module membership (MM) and gene significance (GS). MM is defined as the correlation between the gene's expression profile and the module eigengene, and GS is defined as the correlation between the gene's expression profile and the trait of interest. Each module's hub genes—highly connected genes as potential drivers of co-expression—were selected using a criterion of MM > 0.8 and GS > 0.2, as well as considering the overlap with the previously identified differentially expressed genes. Network visualization was performed with the Cytoscape software (Shannon et al., 2003).

#### 2.4.4 Functional enrichment analysis

Functional enrichment analysis using DAVID Bioinformatics Resources (Sherman et al., 2022) and the R package *clusterProfiler* was applied to annotate the functions of the genes of interest, considering the Gene Ontology (GO) and Kyoto Encyclopedia of Genes and Genomes (KEGG) databases. An adjusted *P*-value (after multiple comparisons correction by the Benjamini-Hochberg method) of < 0.05 was used as the threshold, and the genes participating in more than one pathway were noted.

#### 2.4.5 Correlation evaluation between AD-group's RNA profile and clinical metrics

Spearman's Rank Correlation was performed using R between the normalized counts of differentially expressed genes and two clinical metrics, MMSE and MTA scores, to elucidate the relationship between the notable transcripts and AD pathology. MMSE is a clinical screening questionnaire to evaluate the cognitive performance of potent cognitive-declined subjects (Folstein et al., 1975). Meanwhile, the MTA-score is a clinical metric that quantifies medial temporal lobe atrophy by calculating the width of the choroidal fissure, temporal horn, and height of the hippocampal formation via MRI (Scheltens et al., 1992). After calculating, there would be four levels of atrophy, ranging from 1 to 4. Due to the loss of medical records in the AD cohort, only six MTA records and seven MMSE records were included in the correlation analysis with the respective transcriptomic counts of 136 significant markers. After running the correlation evaluation, significant correlations were plotted and discussed (*p*-value < 0.05). The raw counts of the kept genes were also subjected to the variance stabilizing transformation (vst) to normalize the counts with a constant variance across samples (Zwiener et al., 2014). The vst-data were then utilized to evaluate the dependency of the examining genes on the APOE-genotype from Section 3.

## 3 Results

### 3.1 Sample collection summary, detection of APOE-ε4 allele

Ten plasma samples were successfully collected per cohort together with the subjects' medical records. The samples were stored for 6 months prior to experiments, and the average RNA integrity (RIN) index was 1.79 ± 2, with three samples concluded to be completely free of tissue debris (Supplementary Data 2). In addition, three MMSE records and four MRI records of the AD cohort were missing and henceforth excluded from the correlation analysis. The remaining AD cohort comprised two moderate cases (MMSE 15–20), three moderately severe cases (MMSE 10–14), and two severe cases (MMSE below 10). As mentioned, collected samples were also subjected to APOE-genotyping, which revealed three CNC subjects and seven AD subjects carrying the *APOE-*ε*4* allele (Supplementary Data 1). Discussed details are summarized in Table 2.

**Table 2:** Demographic information of collected samples.

	**Control group (*n* = 10)**	**AD group (*n* = 10)**
Age (years, mean ± SD)	63.6 ± 3.98	67.6 ± 7.82
Sex (male/female)	6/4	3/7
MMSE (maximum score 30)	≥27 (*n* = 10)	Moderate AD: 15–20 (*n* = 2) • Moderately severe AD: 10–14 (*n* = 3) • Severe AD: < 10 (*n* = 2) • Undefined (*n* = 3)
APOE-ε3/ε4	7/3	3/7

**Figure 2:**
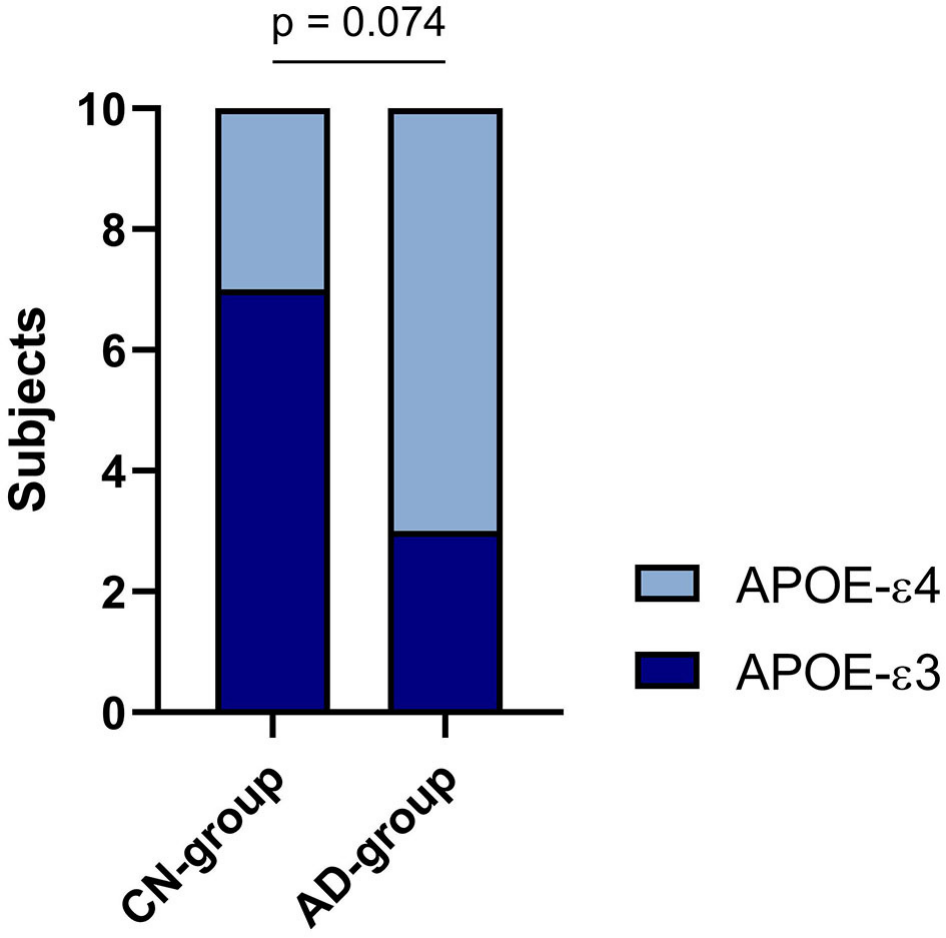
APOE-ε3/ε4 distribution in two cohorts.

As shown in Table 2 and Figure 2, 30% of the CN-cohort and 70% of the AD-cohort carry the *APOE-*ε*4* allele, which is associated with the risks of getting AD. However, chi-square test results revealed an insignificant contribution of the *APOE-*ε*4* allele to the AD diagnosis (*p* = 0.074). The relative risk of getting Alzheimer's found between the *APOE-*ε*4* carrier and non-carrier is 2.4.

### 3.2 Differences in control- and AD-groups' RNA profiles

**Figure 3:**
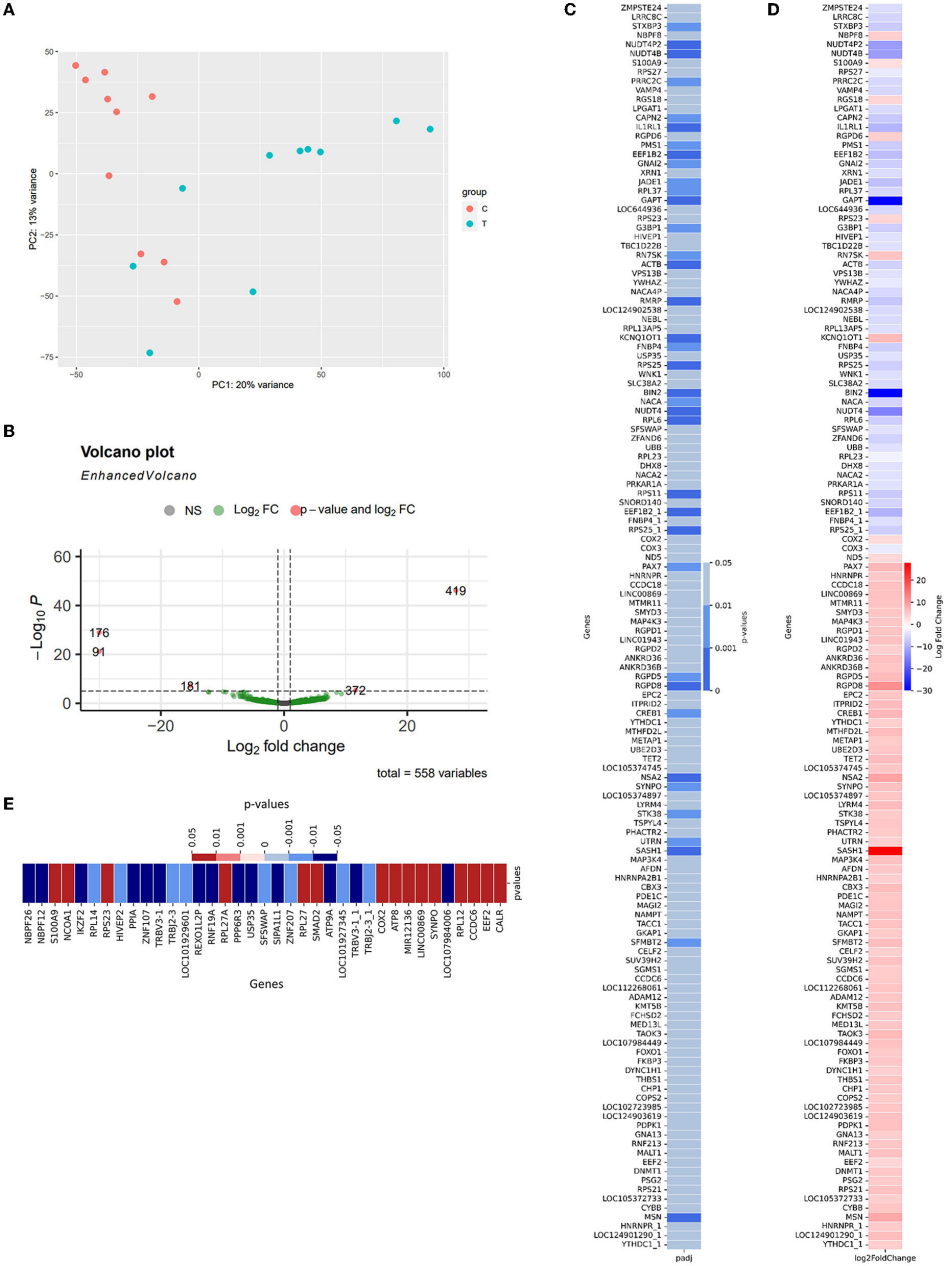
Summary of differential expression results **(A)** PCA plot for similarity clustering; **(B)** Volcano Plot expressed the correlation between the log2foldchange and the adjusted *p*-value of 533 detected genes [gray—*p* > 0.05, green—*p* < 0.05; orange—*p* < 0.0001 and abs(log2foldchange) > 20]; **(C)** The adjusted *p*-value of 88 significant differentially expressed genes; **(D)** Log2foldchange of these genes as AD-CNC; **(E)** The adjusted *p*-value of 37 genes that showed a significant relationship with the existence of APOE-ε4 in the genome (red, positive correlation; blue, negative correlation).

To compare the expression between two cohorts to detect potential diagnosis biomarkers and provide insights about AD pathology within the Vietnam population, differential Expression—DE analysis was conducted according to the described protocol (Section 2.4.2). In brief, the samples collected from two included cohorts exhibit distinctive traits that can be observed as two independent clusters (CNC: orange; AD: green, Figure 3A). 136 significantly differentially expressed genes (DE genes) were identified from 581 detected genes (Supplementary Datum 3, 4; Figure 3B, green dots), with five genes labeled as extremes with *p* < 10^−5^ and abs(log2foldchange)^1^ > 10 (Figure 3B, orange dots). Within 136 significant DE genes (Figure 3C), 19 genes were detected with the highest level of significant difference^2^ between the two cohorts (*p* < 0.001^***^): *SASH1, BIN2, GAPT, NUDT4, RGPD8, EEF1B2_1, IL1RL1, NUDT4P2, NUDT4B, RPS25_1, RPS25, MSN, RPS11, ACTB, RMRP, NSA2, KCNQ1OT1, EEF1B2, RPL6*. There were 18 genes detected with moderately significant differences between the two cohorts (*p* < 0.01^**^): *RGPD5, STXBP3, JADE1, CAPN2, GNAI2, RPL37, RN7SK, NACA, PMS1, FNBP4, PRRC2C, G3BP1, CREB1, SFMBT2, PAX7, SYNPO, UTRN, STK38*. The rest of the list was detected at a low significance level (*p* < 0.05^*^). On the other hand, *GAPDH*—the reference gene, showed an insignificant difference between the two cohorts (*p* = 0.7436). Within the 136 DE genes, there were 84 upregulated genes and 52 downregulated genes (Figure 3D) in the AD group compared to the CNC group. The expression level of *BIN2, GAPT, and NUDT4* decreased significantly in the AD cohort (log2foldchange < −10), with *SASH1 and RGPD8* expressing a notable upregulation in the AD cohort (log2foldchange > 10) (Figures 3B, D) compared to the CNC cohort. The dependency test between the discussed APOE-genotype and the genetic expression level of the DE-genes also revealed 37 genes with their expression level depending on the existence of the *APOE-*ε*4* allele. In detail, the expression level of 15 genes increased significantly when subjects' genomes carried the *APOE-*ε*4* allele (*p* < 0.05), while 21 genes got their expression level deduced (Figure 3E).

**Figure 4:**
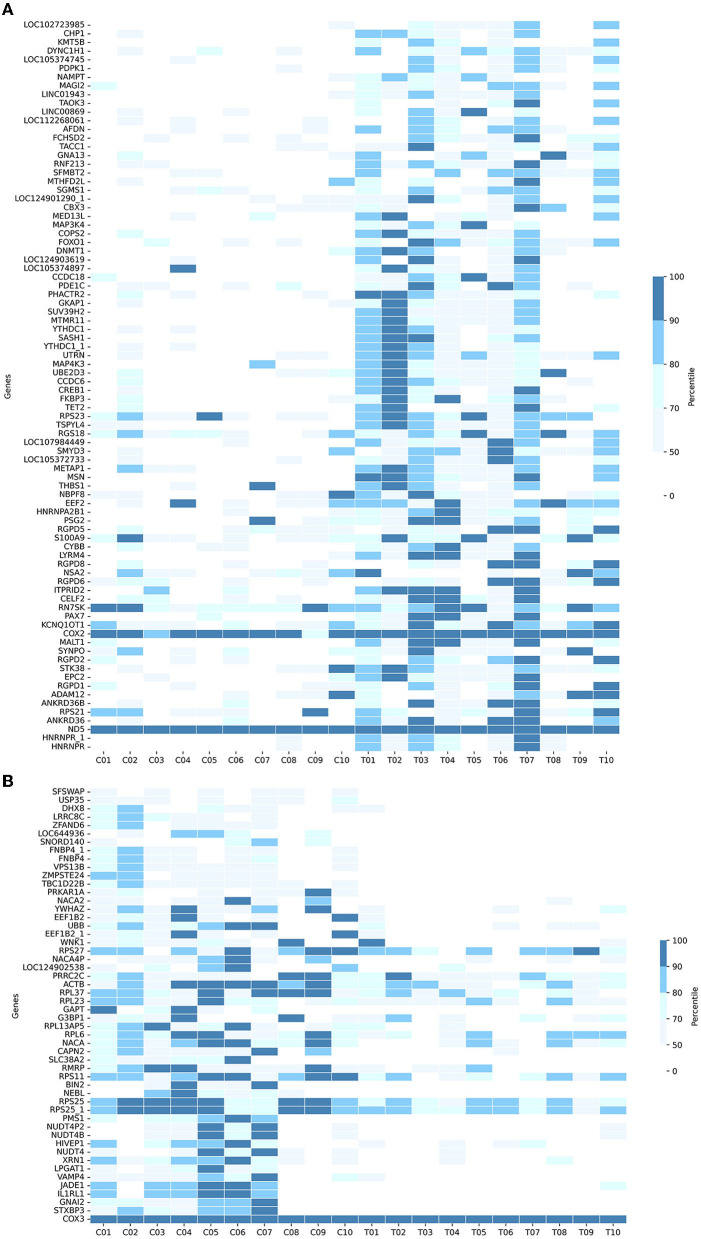
Heatmaps showing the cumulative counts after normalization of the **(A)** upregulated genes and **(B)** downregulated genes in 133 DE-genes (AD-CNC). The scale is divided as the percentile of the counts.

In detail, Figure 4A shows the normalized transcript counts per upregulated gene per subject in two cohorts. In layman's terms, high normalized counts indicate a higher gene expression level in an individual subject and vice versa. 132/840 (15.71 %) of the AD group's records showed counts beyond the 90th percentile of the counts, 4.3 times higher compared to 31/840 (3.690%) of the CNC group. The upregulation was most obvious in the *SASH1* gene as 40% of AD-cohort exceeded the 80th percentile while the CNC-cohort exhibited undetectable counts. This trend resembles *MSN*, where half of the AD cohort passed the 80th percentile while the CNC cohort yielded extremely low to undetectable counts. Considering the downregulated genes (Figure 4B), 384/520 records (73.85 %) of the AD group showed counts below the 50th percentile, which is 2.7 times higher than CNC records with 139/520 CNC records (26.73%). Notably, considering *BIN2 and GAPT* genes, 50% of the CNC group exceeded the 50th percentile threshold, while all of the AD groups did not reach the threshold. The normalized counts of the genes, particularly *BIN2, GAPT, and SASH1*, were consistent with the log2foldchange and the adjusted *p*-values discussed previously.

### 3.3 Co-expression network of AD-related genes

#### 3.3.1 Three modules of interest were identified through network construction and phenotypic correlation

**Figure 5:**
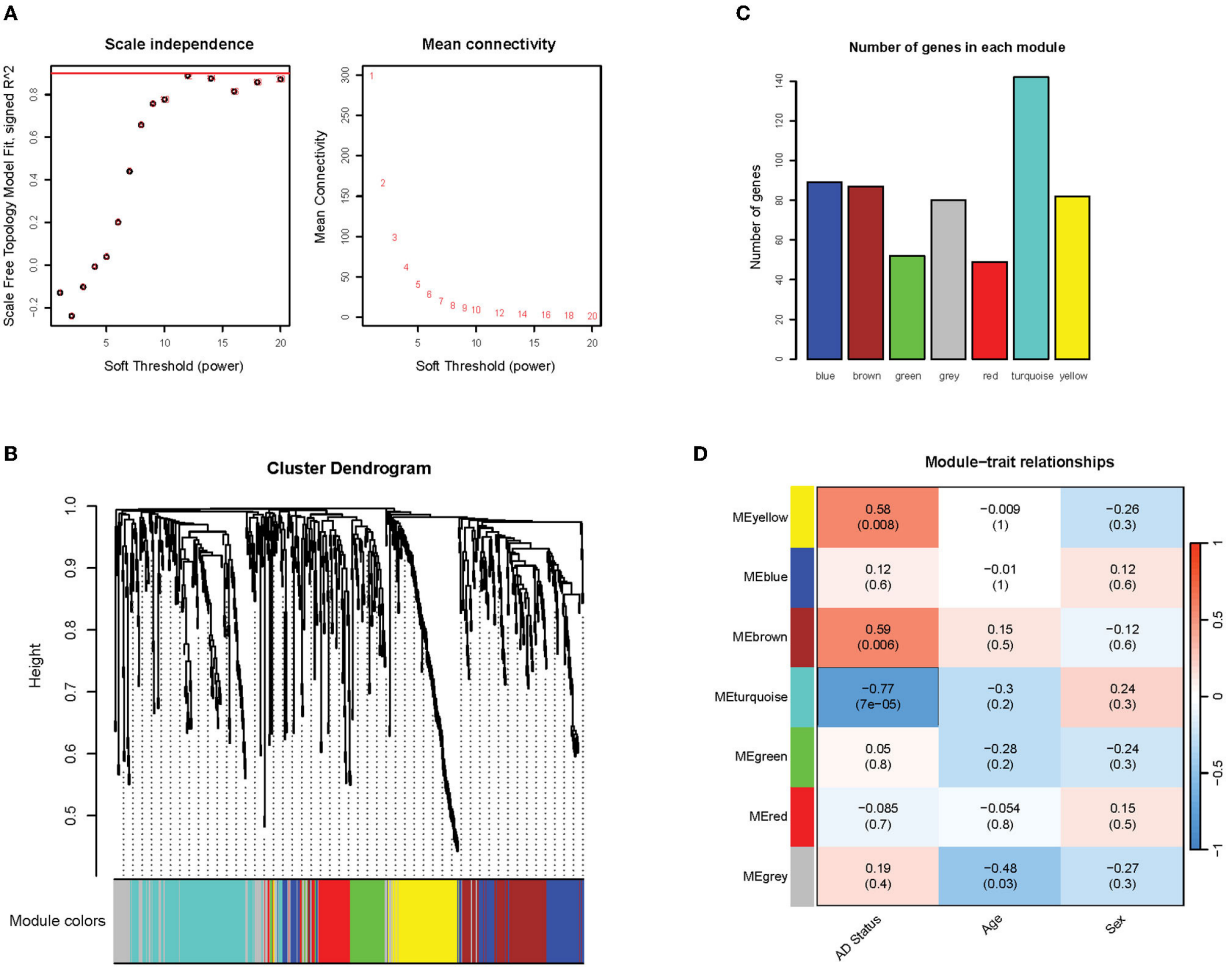
Overview of network construction and module identification. **(A)** Graph of the soft threshold power vs. scale independence and mean connectivity. The red line indicates the scale-free topology fit threshold of *R*^2^ = 0.9; **(B)** Hierarchical clustering dendrogram of the modules. Six modules were detected and assigned arbitrary colors: *blue, brown, green, red, turquoise, and gray* (the *gray* module contains genes that were not clustered into any modules); **(C)** The number of genes in detected modules; **(D)** Pearson correlation coefficients between module eigengenes and phenotypic traits. *P*-values are in parentheses.

A weighted co-expression network on the filtered gene expression matrix was conducted to identify clusters of co-expressed genes. Firstly, it was found that the soft thresholding power of 12 met the scale-free topology fit criteria of R^2^ = 0.9, which ensured the optimal scale-free property (Figure 5A, left) and mean connectivity (Figure 5A, right) of our resulting network. The scale-free property of the network is a key assumption of the WGCNA package to produce biologically meaningful networks (Zhang and Horvath, 2005). The correlation and adjacency matrix was then constructed according to the chosen threshold. After performing hierarchical clustering and dynamic tree cutting on the correlation matrix, six modules of co-expressed genes were obtained, which were assigned arbitrary color names by WGCNA for reference (Figures 5B, C). The number of genes in each co-expression module ranged from 50 to 90 genes, with the exception of the *turquoise* module that contained 142 genes (Figure 5C). To identify modules of particular relevance to AD, we then correlated the expression of the identified modules (using the WGCNA-defined module eigengene measure) with phenotypic variables such as Alzheimer's disease status, age, and sex. We found three modules (*brown, yellow*, and *turquoise*) that correlated significantly with Alzheimer's disease status (*p* < 0.05; *R*^2^ > 0.5) (Figure 5D). This result indicates that these gene modules are likely to be closely associated with and possibly play important roles in Alzheimer's disease. The *brown* and *yellow* modules exhibited a positive correlation with Alzheimer's status (0.58 and 0.59, respectively), while the *turquoise* module exhibited a negative correlation (−0.77) (Figure 5D). We found no statistically significant association between the modules and potentially confounding variables such as patient age or sex.

#### 3.3.2 Functional enrichment analysis and intramodular analysis identify biological function associations and hub genes behind three noted modules

**Figure 6:**
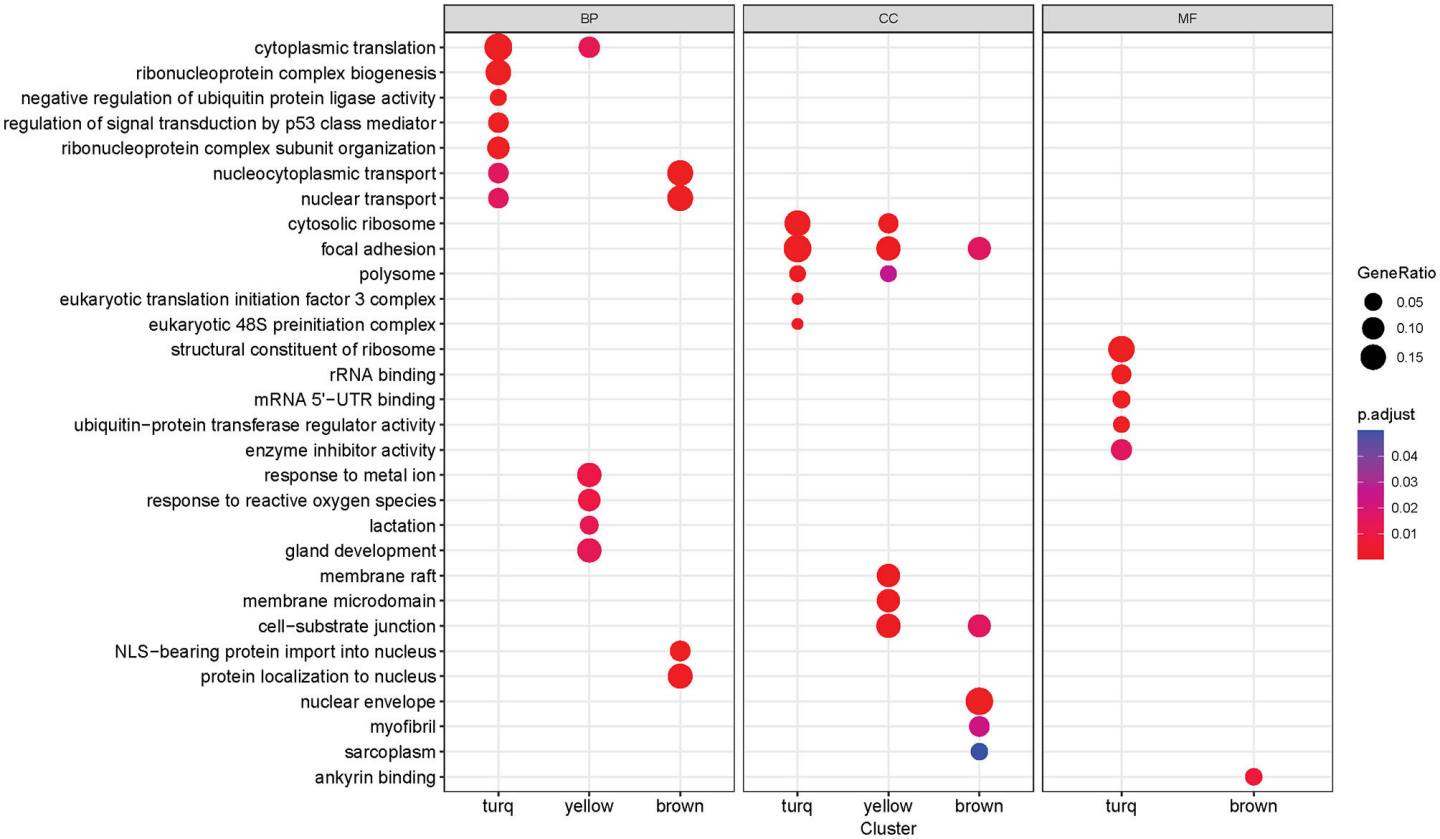
Gene Ontology enrichment analysis results of each co-expression module (*turquoise, yellow, brown*). Each column block represents a GO sub-ontology (BP, biological process; CC, cellular component; MF, molecular function). The dot size represents the gene ratio between genes in the module/gene in the GO set. The dot color represents the Benjamini-Hochberg adjusted *p*-value of the enrichment (only significant terms with *p*-value < 0.05 are shown).

Functional enrichment analysis was performed on three significant modules from the last analysis, with the Gene Ontology (GO) database, to gain insights into the functions of each module as a whole. Many significantly enriched GO terms were detected for all three GO sub-ontologies across the three modules (Benjamini-Hochberg adjusted *p*-value < 0.05), and each module contained a distinct set of significant functional enrichments (Figure 6). The *turquoise* module contained the most significant GO:Biological Process (GO:BP) terms with the highest enrichments regarding cytoplasmic translation and ribonucleoprotein complex-related processes that include biogenesis and subunit organization. The *brown* module is primarily enriched in the nuclear transport, nuclear-cytoplasmic transport, and protein localization processes, while the most prominent GO:BP terms in the *yellow* module are responses to metal ion and reactive oxygen species, as well as gland development and lactation. There were several overlapping enrichments between the modules in the Cellular Component sub-ontology (GO:CC). Focal adhesion was found to be enriched in all three modules, with the gene ratio highest in the *turquoise* module. Another term, cell-substrate junction, was also present in both *brown* and yellow modules. The yellow module was distinguished by a set of significant enrichments related to membrane components such as membrane raft and microdomain, while the most notable GO:CC term in the *brown* module was the nuclear envelope. The Molecular Function sub-ontology (GO:MF) was primarily significant in the *turquoise* module, containing functions related to ribosomes such as structural constituent of the ribosome and rRNA binding. Other significant terms are related to mRNA binding in the untranslated region and enzymatic regulation activity. The *brown* module contained a single significant term related to ankyrin binding.

**Figure 7:**
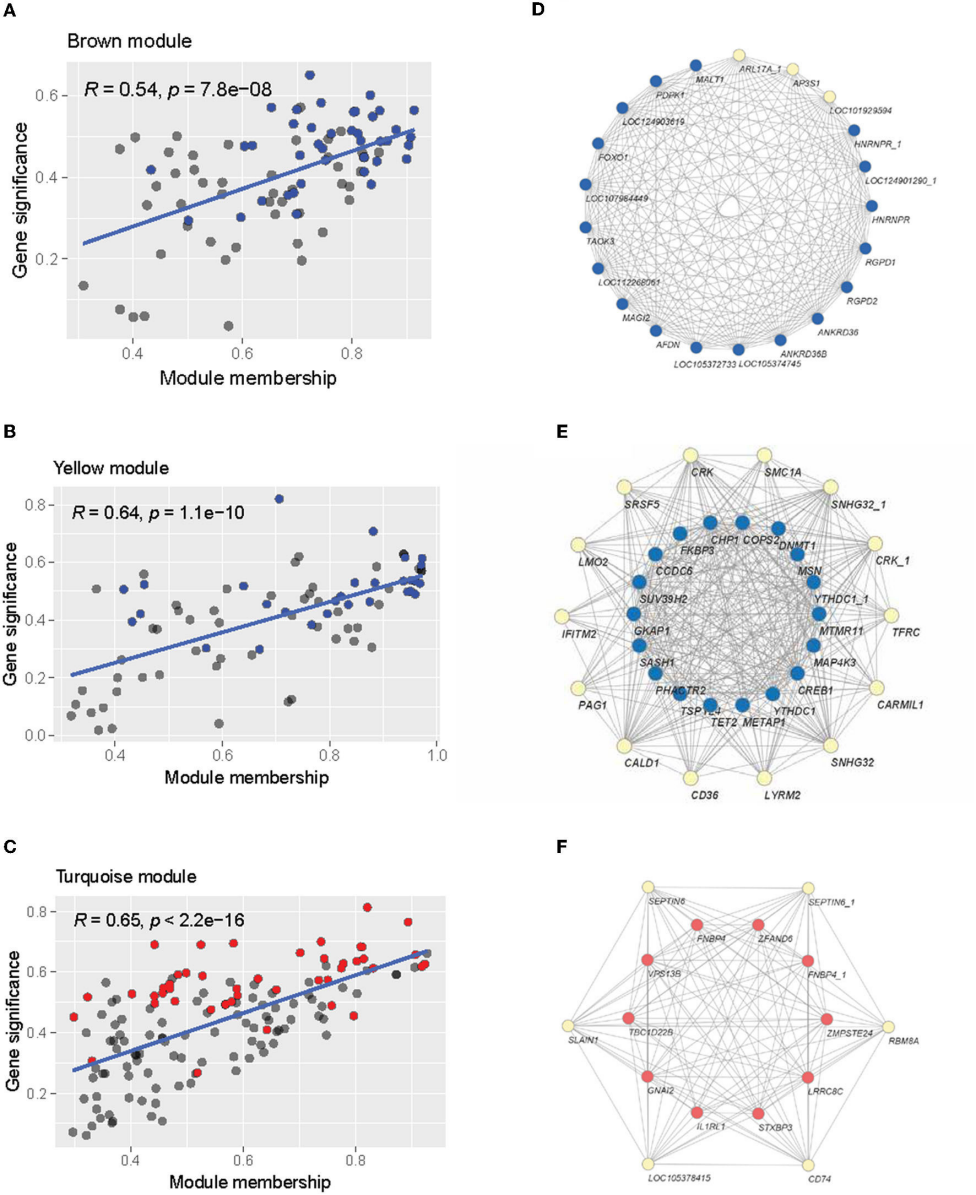
Intramodular analysis. **(A–C)** Scatterplot of module membership vs. gene significance for Alzheimer's disease of the *brown, yellow*, and *turquoise* modules. Individual gray points represent genes in the module. Blue points indicate upregulated genes, and red points indicate down-regulated genes. **(D–F)** Network visualization of the hub genes in the *brown* **(D)**, *yellow* **(E)**, and *turquoise* **(F)** modules. Blue nodes indicate upregulation, while red nodes indicate downregulation.

We then examined the composition of these modules of interest regarding module membership (MM) and gene significance (GS). A higher MM value for a particular gene indicates high connectivity within the module, and a higher GS value indicates a high correlation with the trait of interest (Alzheimer's disease status in this case). Within each module, there is a significant positive correlation (*brown* module: *R* = 0.54, *p* < 0.05; *yellow* module: *R* = 0.64, *p* < 0.05; *turquoise* module: *R* = 0.65, *p* < 0.05) between MM and GS, indicating that the highly connected genes in the module are also significantly associated with Alzheimer's disease status (Figures 7A–C). The *brown* and yellow modules—which were positively correlated with AD status—contained many of the upregulated genes identified through the previous differential expression analysis (Figures 7A, B), while the negatively correlated *turquoise* module contained many of the downregulated genes (Figure 7C). Many of the differentially expressed genes also tended to have high MM and GS measures. From the intramodular analysis, the hub genes—highly connected genes within a module—were selected with the criteria of having an MM value >0.8. Examining these genes, we found that the majority of hub genes within the co-expression network were previously identified as differentially expressed in the previous section (Figures 7D–F). In the *brown* module, 19 out of 22 hub genes were upregulated. The yellow module contained 32 hub genes, of which 18 were upregulated. Finally, the *turquoise* module contained 16 hub genes, of which 10 were downregulated. These overlapping genes were identified as potential candidate genes for further analysis, as they were both highly connected genes and significantly correlated with Alzheimer's disease.

#### 3.3.3 Functional annotation revealed genes that resided in neuronal components and their roles in neuronal activities and immune responses

**Figure 8:**
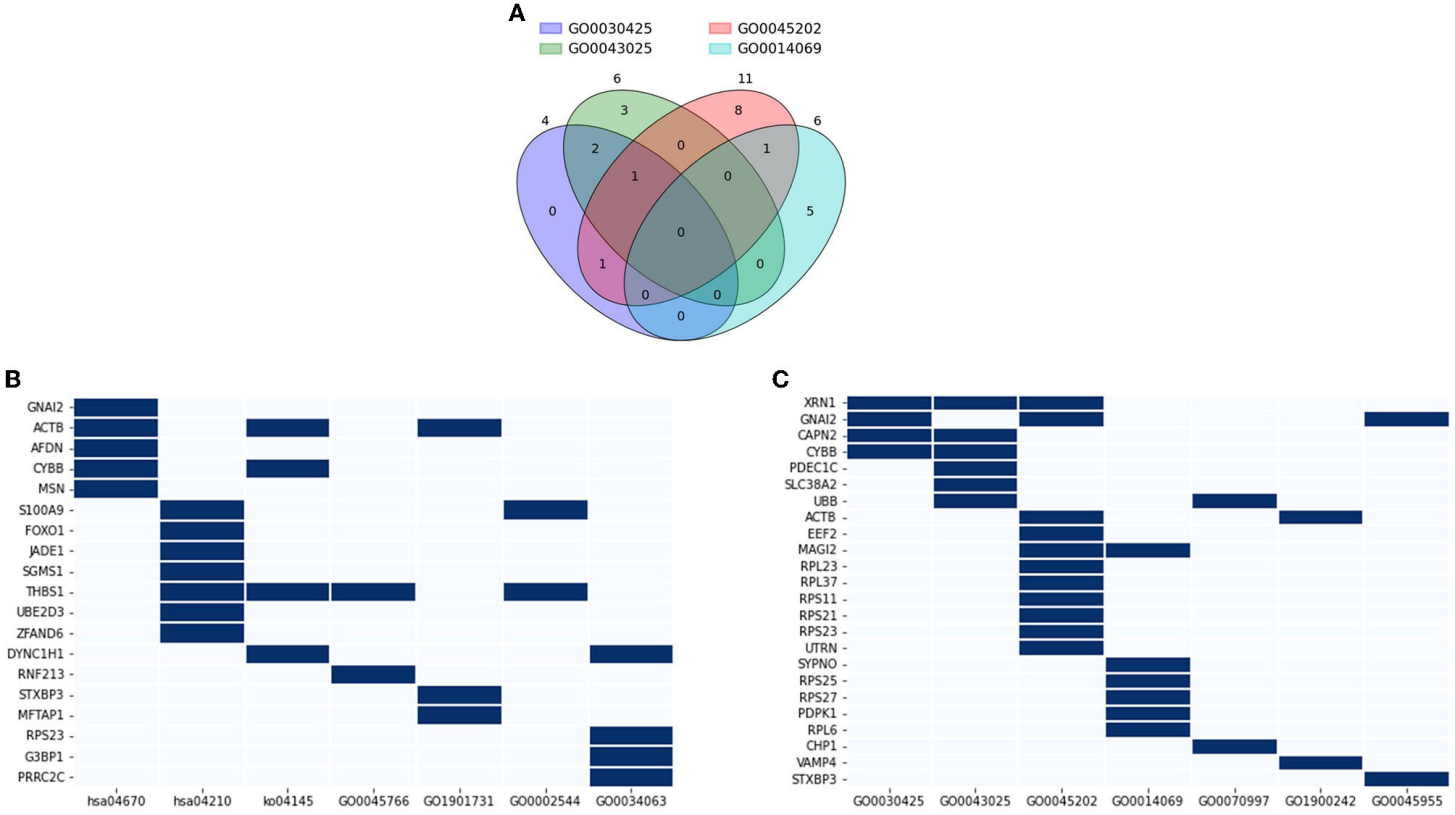
Functional annotation of neuronal and immune-related genes. **(A)** Venn diagram showing a number of overlapping genes that are expressed in four neuronal components: GO0030425 (dendrite), GO0043025 (neuronal cell body), GO0045202 (synapse), GO0014069 (postsynaptic density); **(B)** Genes that highly associated with neuronal activity GO0070997 (neuronal death), GO1900242 (regulation of synaptic vesicle endocytosis), GO0045955 (negative regulation of calcium-ion dependent exocytosis), and four neuronal components; **(C)** Genes that highly associated with immune response and angiogenesis hsa04670 (leukocyte transendothelial migration), hsa04210 (apoptosis), ko04145 (phagocytosis), GO0045766 (positive regulation of angiogenesis), GO1901731 (platelet aggregation), GO0002544 (chronic inflammation), and GO0034063 (stress granule assembly).

Functional annotation analysis using the DAVID was conducted to interpret the role of the DE genes in AD pathology and related pathways. 14 databases were included, which showed a certain level of significance in the correlation between the evaluating gene and the referred function: GO0030425 (*p* = 0.089), GO0043025 (*p* = 0.045), GO0045202 (*p* = 0.00056), GO0014069 (*p* = 0.012), GO0070997 (*p* = 0.066), GO1900242 (*p* = 0.045), GO0045955 (*p* = 0.024), hsa04670 (*p* = 0.0053), hsa04210 (*p* = 0.038), ko04145 (*p* = 0.069), GO0045766 (*p* = 0.1), GO1901731 (*p* = 0.045), GO0002544 (*p* = 0.053), and GO0034063 (*p* = 0.05). Based on the GO_CC (cellular components) database alone, DAVID annotated 21 genes out of the DE genes (15.44 %) that are expressed in neuronal components. Within this geneset, *XRN1* is expressed in three out of four investigated components, namely dendrite, neuronal cell body, and synapse (Figure 8A). Four genes, *GNAI2, CAPN2, CYBB*, and *MAGI2* were traced to express in two components. The rest of the considered gene sets were found in one neuronal component by previous studies. Expand the reference database to the 14 GO_BP and KEGG_pathway databases mentioned at the starting point of this section, three out of 21 discussed genes—*XRN1, UBB, and ACTB*, were found to also participate in the neuronal activity, including neuron death, calcium-dependent exocytosis and synaptic vesicle endocytosis (Figure 8B). Another three genes, including *CHP1, VAMP4, and STXBP3*, were noted to participate in the mentioned pathways yet did not have a significant association with the discussed components in Figure 8A. On the other hand, 19 genes were noted to be a node in seven concerned immune response pathways, namely positive regulation of angiogenesis, leukocyte transendothelial migration, phagocytosis, apoptosis, platelet aggregation, chronic inflammation, stress granule assembly (Figure 8C). Five genes out of the geneset play a link in two or more pathways. Notably, *THBS1* was involved in angiogenesis, phagocytosis, apoptosis, and chronic inflammation. Overall, it can be noted that 38 out of the DE genes (27.94%) were found to be involved in either neural activities or immune response, with five genes enrolled in both categories, namely *STXBP3, ACTB, RPS23, CYBB, and GNAI2*. Considering the high fraction of the DE genes involved in the two AD-related biological pathways, particularly the five overlapping genes, the genes discussed in this section can be promising tools to study AD-pathology and forecast the disease progression as well as the probable complications.

### 3.4 Correlation between the AD group's RNA profile and AD clinical metrics

**Figure 9:**
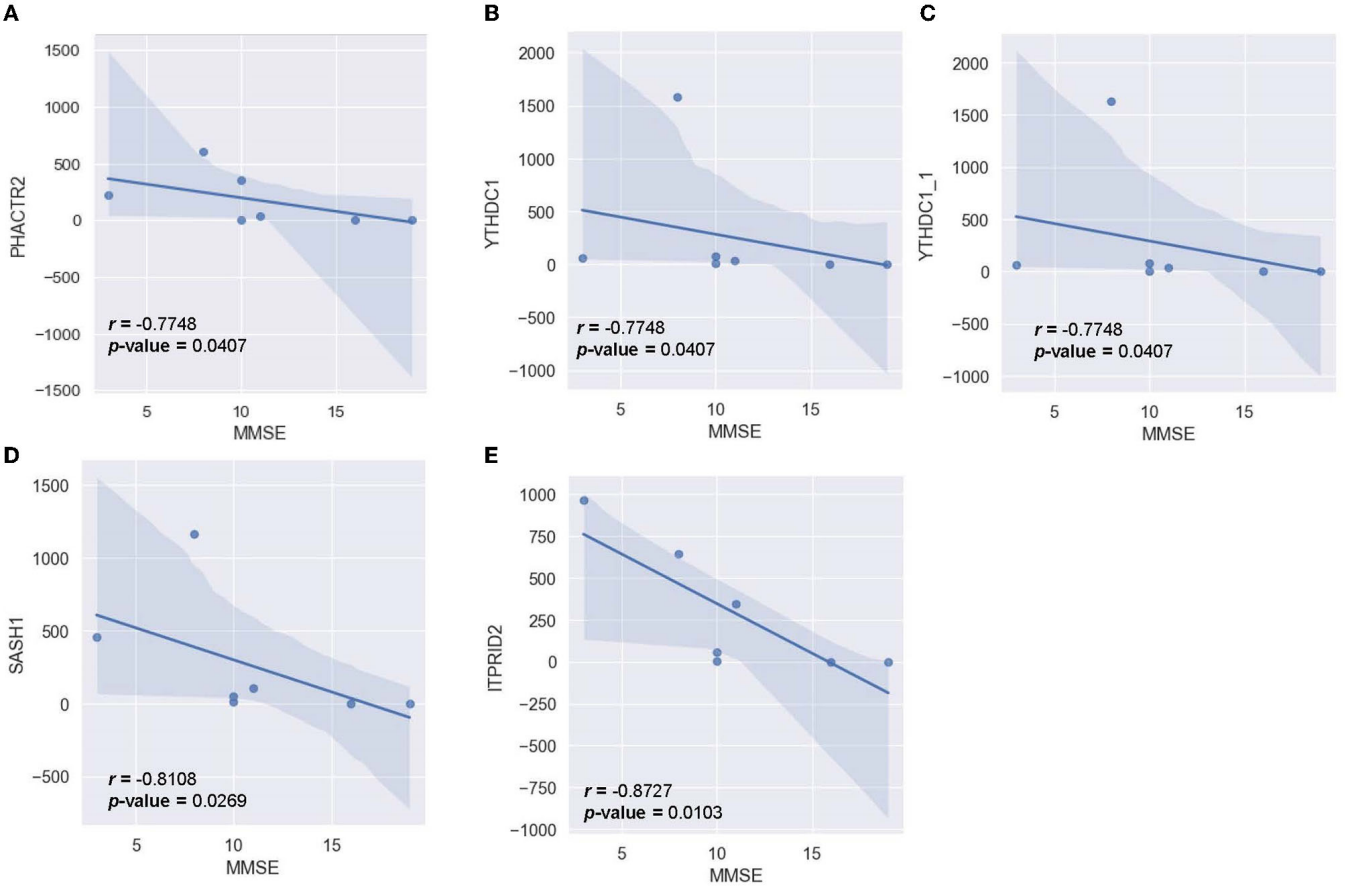
Correlation between MMSE-score and the normalized transcript counts (median of ratios) in five genes **(A)** *PHACTR2*, **(B)** *YTHDC1*, **(C)** *YTHDC1_1*, **(D)** *SASH1*, **(E)** *ITPRID2*.

To determine the relationship between significant alternating plasma biomarkers and subjects' cognitive performance, Pearson correlation was conducted between the MMSE-score of the AD cohort (*n* = 7) and the respective transcriptomic counts of 136 significant markers. Five genes were found to be inversely correlated with the MMSE scores, including *PHACTR2, YTHDC1, YTHDC1_1, SASH1, and ITPRID2* (Figure 9). The detected trend indicated that as the transcriptomic level of these genes increased, subjects' MMSE levels were significantly reduced. The correlation is most notable in *ITPRID2* (*R* = −0.873; *p* = 0.0104) and *SASH1* (*R* = −0.811; *p* = 0.0269) (Figures 9D, E, respectively).

**Figure 10:**
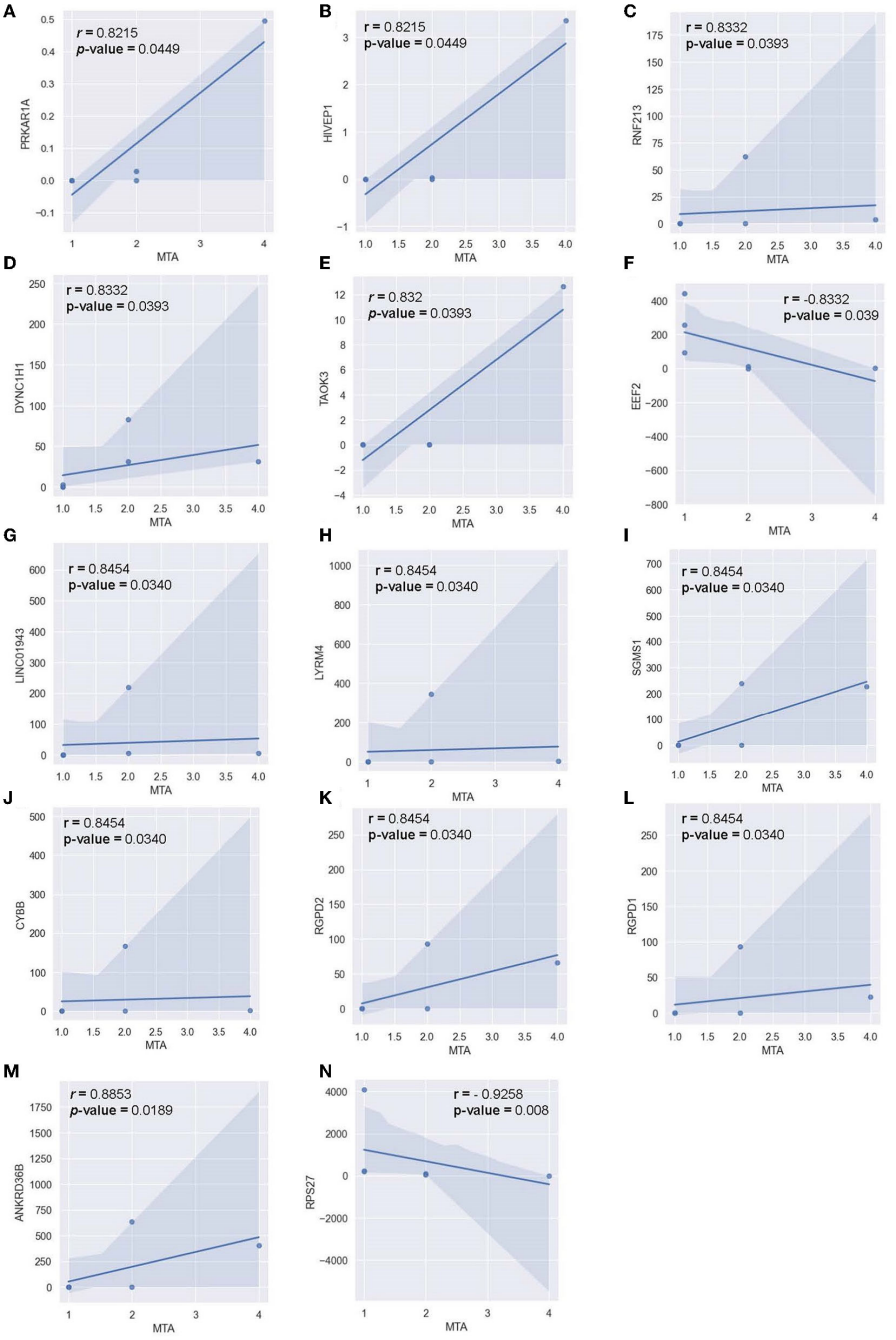
Correlation between MTA-score and the normalized transcript counts (median of ratios) in 14 genes **(A)** *PRKAR1A*, **(B)** *HIVEP1*, **(C)** *RNF213*, **(D)** *DYNC1H1*, **(E)** *TAOK3*, **(F)** *EEF2*, **(G)** *LINC01943*, **(H)** *LYRM4*, **(I)** *SGMS1*, **(J)** *CYBB*, **(K)** *RGPD2*, **(L)** *RGPD1*, **(M)** *ANKRD36B*, **(N)** *RPS27*.

Pearson correlation was also conducted between the MTA-score of the AD-cohort (*n* = 6) and the respective transcriptomic counts of significant markers to determine the relationship between significant alternating plasma markers and subjects' hippocampal atrophy (Figure 10). Compared to the MMSE score, the MTA score showed significant correlations with multiple markers in a more diverse pattern. In detail, 14 genes were found to correlate significantly with the said index, with 12 genes being positively correlated and two genes being negatively correlated. Within the positively-correlated plasma markers, *ANKRD36B* showed the most significant association with the MTA score (Figure 10M, *R* = 0.885 and *p* = 0.0190), indicating that as the marker increased its expression level, the atrophy elevated in subjects' brains accordingly. In contrast, *RPS27* stood out between two negatively correlated markers (Figure 10N, *R* = −0.926 and *p* = 8.1 x 10-3), implying the reduced expression level of this marker would be associated with the expansion of the atrophy in subjects' brains.

## 4 Discussion and conclusion

Up-to-date, blood-based cfRNAs analysis is a promising approach for the diagnosis of AD at the early stage compared to other methods (such as MRI, PET, or MoCA questionnaire), which objectively and quantitatively reveals the progression of AD pathology and solves the financial conundrum at LMICs. This preliminary study examined the difference between the cfRNA profiles of two Vietnamese cohorts (Alzheimer's and normal control), which recruited 10 participants per group. All plasma samples from two cohorts were successfully collected, revealing 10 *APOE-*ε*4* carriers (three subjects in the CN cohort; seven subjects in the AD cohort), confirming the AD prevalence of this allele was twice the ε*3* allele. In addition, the medical records of six MTA and seven MMSE scores were collected for Spearman's Rank Correlation evaluation to find promising prognostic biomarkers.

DE analysis was performed to further investigate the promising biomarkers for AD diagnosis and has identified 136 differentially expressed genes from 581 input genes (Supplementary Datum 3, 4; Figure 3B, green dots), 84 of which were upregulated, and 52 were downregulated (Figure 4). To ensure the reliability of the results, we first compared the expression of the *GAPDH*—a housekeeping gene, between two cohorts, and there was no significant difference (Supplementary Data 2). Three outstanding genes were noted with outstanding log2foldchange and adjusted *p*-value (Figure 3B). Also, from the collected cfRNA dataset, three modules of co-expressed genes were detected. These modules were found to be involved in various biological processes and functions through enrichment analysis. The overlapping genes between the groups of highly connected hub genes and previously identified DE genes were highlighted in the co-expressed network as outstanding candidates for further analysis (Figure 7). Thirty-eight DE genes were also noted to be involved in the neural activities and immune responses, and five of which are involved in both pathways. This result implicated a high potential of the detected genes as clinical markers and tools to study the pathogenesis of AD (Figure 8). In addition, there were five genes having a significantly inverse trend with MMSE score (Figure 9), three of which were hub genes classified in the *yellow* module, *YTHDC1, PHACTR2, and SASH1*. On the other hand, the correlation with MTA scores showed more diversity compared to MMSE metrics, with 14 DE genes denoted with a high correlation with the metric. In detail, the greatest-positive trend was identified in the *ANKRD36B* gene (Fig.M, *R* = 0.885 and *p* = 0.0190), and the significantly negative association indicated in the *RPS27* gene (Fig.N, R = −0.926 and *p* = 8.1 × 10–3) (Figures 10M, N).

The co-expression analysis revealed three distinct clusters of co-expressed cfRNA transcripts that were strongly correlated with AD status (Figure 5D). Through enrichment analysis, we found that the enriched functions of these clusters are highly relevant to AD pathogenesis and progression (Figure 6). Notably, all three clusters contained many genes related to focal adhesions. It has been reported that focal adhesions participate in various pathways that regulate amyloid-beta signaling, eventually leading to neuronal cell death (Caltagarone et al., 2007). Each module was also involved in unique functions. The *yellow* module was enriched in biological process terms regarding response to metal ions and reactive oxygen species. A recent study (Chen et al., 2023) hypothesized that the dyshomeostasis of metal ions (e.g., iron, copper, zinc, and calcium) in the brain of AD patients is a possible cause for several AD-related pathologies. Specifically, the imbalance of metal ions can lead to the overproduction of amyloid beta, neuroinflammation, and tau hyperphosphorylation. Furthermore, this disruption in metal ion balance leads to increased oxidative stress and the production of reactive oxygen species (ROS), which has been associated with neuronal damage (Wang et al., 2020). This functional finding is also corroborated by Toden et al., who found a cluster of genes enriched in calcium signaling in Alzheimer's plasma cfRNA transcripts. Finally, in the *brown* module, the most notable terms were related to nuclear transport, nuclear-cytoplasmic transport, and protein localization into the nucleus. It has been suggested that altered nuclear transport and protein mislocalization are possible mechanisms for the development of neurodegeneration (Sheffield et al., 2006). This disruption is thought to be caused by tau proteins, a key hallmark of Alzheimer's disease. Tau proteins can interact with nucleoporins in the nuclear pore complex, causing mislocalization, blocking nuclear import/export, and eventually leading to neuronal death (Eftekharzadeh et al., 2018). These Alzheimer-linked functional associations point to the significance of our detected modules, which can be a potential avenue for further research.

Within each co-expressed module were multiple hub genes with high intramodular connectivity (Figure 7). These hub genes are likely to influence the expression of other genes in the module, thus acting as key drivers in the module's biological functions and pathways (Langfelder and Horvath 2008). Of special interest are hub genes that are also found to be differentially expressed between AD and healthy controls in our study (significant hub genes) since these genes are likely to be highly influential and relevant to AD. Notably, several of these significant hub genes (Figures 7D–F) were previously reported to be associated with Alzheimer's disease or linked with neurodegeneration processes. For example, in the *brown* module, the significant hub gene *RNF213* was found to be associated with Alzheimer's disease in a previous RNA transcriptome study on whole blood from an American cohort (Bai et al., 2014). Two ankyrin-related genes were also centrally located in the *brown* module (*ANKRD36* and *ANKRD36B*), possibly suggesting the involvement of ankyrin-binding pathways in AD. In the *yellow* module, two significant hub genes are related to calcium and iron response (*CREB1* and *TFRC*). *CREB1* is involved in the pathways of calcium signaling, which has been previously implicated in neurodegeneration (Tong et al., 2018). *TFRC* acts as an iron uptake mediator in the central nervous system, and changes in its expression can lead to dyshomeostasis in iron concentrations in the brain (Rouault, 2013). The *yellow* module also contains the hub gene *YTHDC1* that regulates N6-methyladenosine (m6A) RNA methylation, the disruption of which has been associated with increased AD risk (Qiu et al., 2023). Finally, the significant hub gene of interest in the *turquoise* module is the interleukin receptor *IL1RL1*. Multiple studies have reported a mutation in *IL1RL1* that is linked with a decreased Alzheimer's disease risk by reducing circulating ST2 levels (Jiang et al., 2022). Since the *turquoise* module as a whole is negatively correlated with AD status, and *IL1RL1* is dysregulated in our dataset, this points to a possible protective effect of the *IL1RL1* gene in our cohort. In addition, many of the significant hub genes (*YTHDC1, PHACTR2, SASH1, ANKRD36B, RNF213, RGPD2, TAOK3*) were also significantly correlated with MMSE and MTA scores in our cohort, further suggesting their relevance to AD pathogenesis. Overall, these significant hub genes can help shed light on potentially new pathways and interactions and provide guidance for further investigation as biomarkers or therapeutic targets since they are likely to influence the expression of a multitude of genes.

### 4.1 DE genes and neural activities

Functional annotation analysis indicated 21 genes expressed in neuronal components, three of which are also involved in neural activities. Notably, *XRN1* is expressed in three out of four investigated components, namely dendrite, neuronal cell body, and synapse (Figures 8A, B). *XRN1*, together with *STXBP3*, were found to participate in the negative regulation of calcium-ion-dependent exocytosis, which is linked with synaptic transmission by releasing quanta of neurotransmitters (Barclay et al., 2005). Both of these genes are significantly downregulated in the AD cohort (Figure 3D), suggesting a probable increase in neurotransmitter release at the synaptic cleft. In previous reports, the elevation in other neurotransmitters, particularly dopamine, glutamate, and norepinephrine, stimulates cognitive dysfunction in AD patients, along with the deficiency of Acetylcholine (Xu et al., 2012; Bhuvanendran et al., 2018; Mather, 2021; Chen et al., 2022). Besides, *XRN1* has been identified as a risk contributor of late-onset AD previously (Guttula et al., 2012; Rosenthal et al., 2012; Xu et al., 2019), in which the significant deduction of *XRN1* transcripts in the AD cohort can explain the imbalance in neural activities that are associated with AD pathology. Considering neuronal death, two genes in the DE list were found to be associated—*UBB and CHP1* (Figure 8B). While *CHP1* was upregulated in the AD cohort, *UBB* expressed the opposite trait (Figure 3D). *CHP1* is a promoter-encoding gene that facilitates the activities of the sodium/hydrogen exchangers (NHEs) activating neuron death, implying that its upregulation in the AD cohort can be due to increasing neural apoptosis triggered by AD (Song et al., 2019). The reduction in *CHP1* expression level was reported as a prominent treatment for neural injury by promoting axonal outgrowth (Janzen et al., 2018). *UBB*, on the other hand, is involved in the Ubiquitin system that modulates synaptic plasticity and neural homeostasis (Harris et al., 2020). The deficiency in cellular Ubiquitin has been stated to suppress the survival capacity and lead to neuronal death, which is well-aligned with our previous argument that neural apoptosis occurred more robustly in AD patients (Ryu et al., 2008). In brief, the findings from functional annotation fit with previous findings and also support future investigation of not only biomarkers but also therapeutics targets for AD, considering the *XRN1, CHP1*, and *UBB*.

### 4.2 DE genes and immune responses

Immune responses are another aspect that we included in this discussion, considering its bond with AD pathology, both as a probable stimulator and a complication (Webers et al., 2020; Griciuc and Tanzi, 2021). Considering how immune responses can reduce the resilience of the CNS toward the amyloid plaques, immune-related genes have been studied and recommended as risk factors accompanying the *APOE* genotype (Griciuc and Tanzi, 2021). We managed to identify transcripts of *THBS1*, which was noted to be involved in multiple investigated immune-related pathways, namely angiogenesis, phagocytosis, apoptosis, and chronic inflammation. Previous studies have reported an association between the upregulation of *THBS1* and increasing neuroinflammation, which put the CNS in jeopardy (Wang et al., 2023; Yao et al., 2023). This suggests the upregulation observed in the AD cohort implied an increasing inflammatory response that fits with precedent postulations. Knocking out the gene and inhibiting its expression have been studied in animal models as a means to ameliorate inflammatory processes (Qu et al., 2020; Wang et al., 2023). In addition to *THBS1, S100A9* is another gene that takes part in chronic inflammation, as well as apoptosis, and was noted in our study to be upregulated with the existence of the *APOE-*ε*4* allele (Figure 3E). This finding is well-aligned with previous studies, which reported the upregulation of *S100A9* in advancing AD, and the knockdown of this gene can alleviate memory capacity in animal models (Shepherd et al., 2006; Chang et al., 2012; Wang et al., 2014). According to the discussed studies, *THBS1* and *S100A9* appeared to be probable risk factors for AD and a prominent target for molecular therapeutics.

### 4.3 DE genes and clinical metrics

Two out of five genes that are highly correlated with the MMSE score, namely *ITPRID2 and PHACTR2*, participate in the binding process of Actin (Figure 9). Previous studies postulated that cognitive impairment was associated with imbalanced Actin-binding factors, such as increased dephosphorylated cofilin and decreased drebrin (Kojima and Shirao, 2007; Bamburg et al., 2010, 2021). This implied the consistency of our finding and the precedent results, confirming the role of Actin dynamics in cognitive functions. Considering 14 genes professed high correlation with MTA-score, *DYNC1H1, and EEF2* are directly involved in neurodegeneration (KW-0523). Two other genes, *RNF213 and SGMS1*, participate in lipid metabolism, which is involved in neurodegeneration (KW-0443) (Estes et al., 2021). *RGPD1 and RGPD2*, on the other hand, take part in intracellular transport and catalytic activity (GO:0046907; GO:0050790) (Stefanova et al., 2019). Other genes play a role in either nucleic acid repairing process, ionic transport, or protein binding, which put forward a postulation about the probable role of these processes in the structural changes inside the brains of AD patients (Figure 10).

Comparing our detected DE genes to two previous studies profiling the plasma cf-RNA of AD patients (Toden et al., 2020; Fu et al., 2023), we found relatively minimal overlap between the three studies (Supplementary Data 7). The Fu study was a pilot study on a small cohort (*n* = 40) of Chinese AD and healthy patients, while the Toden study was performed on a large cohort of patients in the USA (*n* = 242). While a small number of our DE genes overlapped with the Toden group's result, there were also a significant number of contradictory genes (i.e., upregulated genes that were found to be downregulated by Toden). In addition, the Toden study and Fu study had only one DE gene in common. This indicates significant variability in detected DE genes across different studies and cohorts. Some potential factors include methodological differences between groups, sample size, and geographic variations in gene expression, which calls for further investigation.

In conclusion, this is the first study in Vietnam to collect and evaluate the cfRNA transcriptome from plasma samples of AD patients. With this novel dataset, we employed a combination of differential expression analysis and weighted co-expression network analysis to identify candidate gene transcripts in cfRNA for further studies into diagnostic biomarkers and therapeutic applications. Several candidate transcripts were identified that were differentially expressed, highly connected, well-correlated with AD clinical markers, and relevant to the pathogenesis of AD (*CREB1, YTHDC1, IL1RL1, PHACTR2, ANKRD36B, RNF213)*. These candidate genes from our dataset indicate the potential for plasma cfRNA as an AD diagnostic biomarker and as a tool to elucidate the complex mechanisms of AD. Other transcripts, specifically ones related to immune response like *THBS1* and *S100A9*, were also recorded in our report with similar traits as previous independent studies. This bolsters the potential of further studies targeting the relationship between the immune response and AD pathology, which can be an inspiration for therapeutic studies considering the recent progress of immunotherapy. Owing to plasma's relatively minimally invasive sampling compared to other methods, this approach allows for an accessible window into the dynamic transcriptional alterations of the AD brain, which will hopefully improve our understanding of AD pathogenesis and aid in the development of AD diagnostic tools.

Our pilot study has several limitations owing to its proof-of-concept nature. Considering the sample size of this study is scant, there is a potentially high FDR and compromising comparison tests' power (Liu and Hwang, 2007). Acknowledging that the limited sample size can cast doubt upon the conclusion of significance, the statistical tests utilized in this study were all non-parametric tests, which do not involve the sample size in the hypothesis, except for the correlation tests. Additionally, the algorithms behind the differential expression analysis in the DESeq2 package already included the size factors in their comparisons (Love et al., 2014). Therefore, the conclusions of significance in this study are accurate within the examined range (Table 2). Besides, due to financial restrictions, patients with MCI cannot be included in this study to examine the differences in transcriptomic profile at the early stages of AD pathology. Moreover, our extracted samples had unsatisfactory quality, considering their purity and integrity (Supplementary Data 2). Despite having normalized the sequencing data, there is a probability of deviation occurring in the results. However, the insights obtained from this study can be the foundation for similar studies on larger populations, as well as support a more detailed investigation of highlighted genes and their potential as AD biomarkers and therapeutic targets. In addition, comparing the expression traits between plasma transcriptomic profiles and those of brain tissues is also a potential field of inquiry that can provide further insights into cell-free AD biomarkers.

## Data availability statement

The original contributions presented in the study are publicly available. The raw sequence reads of this study have been deposited at https://www.ncbi.nlm.nih.gov/bioproject/PRJNA1024653.

## Ethics statement

The studies involving humans were approved by University Medical Center, Ho Chi Minh City. The studies were conducted in accordance with the local legislation and institutional requirements. The participants provided their written informed consent to participate in this study.

## Author contributions

TC: Writing – original draft, Conceptualization, Data curation, Formal analysis, Investigation, Methodology, Project administration, Visualization. AL: Conceptualization, Formal analysis, Investigation, Methodology, Project administration, Visualization, Writing – original draft, Writing – review & editing. TTT: Data curation, Resources, Writing – original draft, Writing – review & editing. VH: Formal Analysis, Visualization, Writing – original draft. BP: Investigation, Writing – original draft. TL: Data curation, Writing – review & editing. QN: Data curation, Visualization, Writing – review & editing. TCT: Data curation, Resources, Writing – review & editing. TMT: Data curation, Resources, Writing – review & editing. THNT: Data curation, Resources, Writing – review & editing. TN: Data curation, Resources, Writing – review & editing. HH: Conceptualization, Funding acquisition, Supervision, Validation, Writing – review & editing.

## Footnotes

^1^Absolute value of the Log2foldchange.

^2^According to the APA scale.
