# Supplementary material for: Plasma cell-free RNA profiling of Vietnamese Alzheimer's patients reveals a linkage with chronic inflammation and apoptosis: a pilot study
Source: Front Mol Neurosci. 2023 Dec 21;16:1308610. doi: 10.3389/fnmol.2023.1308610 (PMC10764507; doi:10.3389/fnmol.2023.1308610)
Supplement: Supplementary file 1 [file Table_1.DOCX]

**Supplementary File 1: Genotyping of APOE-E3/E4 alleles**

| **Name** | **Direction** | **Sequence (5’ – 3’)** | **T_m_ (°C)** | **GC%** | **Length (bp)** |
| --- | --- | --- | --- | --- | --- |
| **Allele-specific primers for APOE-E3/E4** | | | | | |
| E3 | F | CGGACATGGAGGACGTGT | 59.6 | 61.11 | 18 |
| E4 | F | CGGACATGGAGGACGTGC | 61.1 | 66.67 | 18 |
| E3m | F | CGGACATGGAGGACGTTT | 57.6 | 55.56 | 18 |
| E4m | F | CGGACATGGAGGACGTTC | 58.6 | 61.11 | 18 |
| Common reverse | R | GCTTCGGCGTTCAGTGATTG | 58.9 | 55.00 | 20 |
| **Positive control primers** | | | | | |
| ACTB-F | F | GACGTGGACATCCGCAAAGAC | 63.6 | 57.14 | 21 |
| ACTB-R | R | CAGGTCAGCTCAGGCAGGAA | 61.3 | 60.00 | 20 |

**Supplementary Table 1:** Sequence and characteristics of the primers used in this study.

|  | **E3** | **E4** | **E3-m** | **E4-m** |
| --- | --- | --- | --- | --- |
| **C1** | x | x | x |  |
| **C2** | x | x | x | x |
| **C3** | x | x | x |  |
| **C4** | x | x | x | x |
| **C5** | x | x | x |  |
| **C6** | x | x |  | x |
| **C7** | x | x | x |  |
| **C8** | x |  |  |  |
| **C9** | x | x | x |  |
| **C10** | x | x | x |  |
| **T1** | x | x | x |  |
| **T2** | x | x | x | x |
| **T3** | x | x | x | x |
| **T4** | x | x | x | x |
| **T5** | x | x |  | x |
| **T6** | x | x |  |  |
| **T7** | x | x | x | x |
| **T8** | x | x | x | x |
| **T9** | x | x | x | x |
| **T10** | x | x | x |  |

**Supplementary Table 2**: Summary of APOE genotyping results. A check indicates a positive detection of the PCR product of that primer. The E3m and E4m primers were used to conclude the presence of the APOE-E4 allele, due to the high presence of possible false positives with the E3 and E4 primer pair.
