# Supplementary material for: Plasma cell-free RNA profiling of Vietnamese Alzheimer's patients reveals a linkage with chronic inflammation and apoptosis: a pilot study
Source: Front Mol Neurosci. 2023 Dec 21;16:1308610. doi: 10.3389/fnmol.2023.1308610 (PMC10764507; doi:10.3389/fnmol.2023.1308610)
Supplement: Supplementary file 7 [file Data_Sheet_6.docx]

**
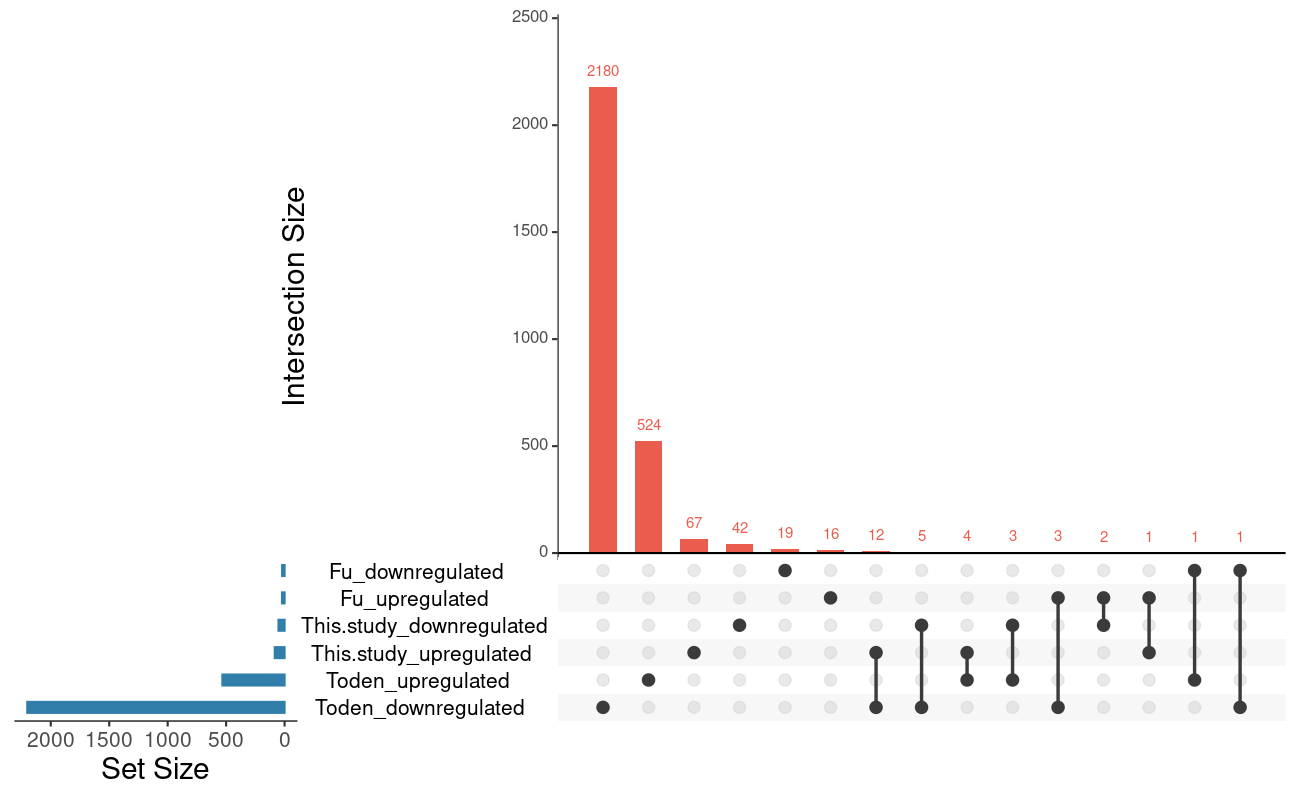
**

**Supplementary Figure 1:** UpSet plot of intersections between upregulated and downregulated genes of different plasma cfRNA studies: this study, Toden et al. (2020), and Fu et al. (2023).
